# Dr. Joseph Haberstro

**Published:** 1898-08

**Authors:** 


DR. JOSEPH HABERSTRO, of Buffalo, died at his home, 769 Genesee street, July 12, 1898, aged 44 years. Dr. Haberstro had suffered from consumption for some time past and during the past few weeks had been steadily growing worse, his condition having been such that no hope was entertained for his recovery. It is supposed that he contracted the disease while serving as resident physician at the Erie County hospital.

Dr. Haberstro was a son of ex-sheriff Joseph L. Haberstro and was a graduate of the medical department of the University of Buffalo, class of 1877; he also studied at Ann Arbor, Mich., and Bellevue Hospital, New York. He had returned from New York City about four weeks before his death, where he had been taking treatment at the Pasteur Institute. Dr. Haberstro was a member of Concordia Lodge of Masons, an Odd Fellow and a member of the Exempt Firemen's Association. The funeral was held at 2 o'clock Friday afternoon, July 15th, under the auspices of Concordia Lodge of the Masonic order and the services were conducted by the Rev. William Grommish, chaplain of the order.

The honorary bearers were Drs. Auel, Storck, Fowler, Phelps, Gott, Martin, Meisburger and Edwin G. S. Miller. The active bearers, members of Concordia Lodge, were: Ernest Bamberg, Charles F. Stern, Peter Braner, Peter Funk, Valentine Frank, Daniel Wander, Jacob Roth and Frank Mauerman. Many of Buffalo's prominent citizens were at the funeral. The burial was in Forest Lawn.

